# Characteristics of the Norwegian Coastal Current during Years with High Recruitment of Norwegian Spring Spawning Herring (*Clupea harengus* L.)

**DOI:** 10.1371/journal.pone.0144117

**Published:** 2015-12-04

**Authors:** Øystein Skagseth, Aril Slotte, Erling Kåre Stenevik, Richard D. M. Nash

**Affiliations:** 1 Institute of Marine Research, Bergen, Norway; 2 Bjerknes Centre for Climate Research, Bergen, Norway; Technical University of Denmark, DENMARK

## Abstract

Norwegian Spring Spawning herring (NSSH) *Clupea harengus* L. spawn on coastal banks along the west coast of Norway. The larvae are generally transported northward in the Norwegian Coastal Current (NCC) with many individuals utilizing nursery grounds in the Barents Sea. The recruitment to this stock is highly variable with a few years having exceptionally good recruitment. The principal causes of recruitment variability of this herring population have been elusive. Here we undertake an event analysis using data between 1948 and 2010 to gain insight into the physical conditions in the NCC that coincide with years of high recruitment. In contrast to a typical year when northerly upwelling winds are prominent during spring, the years with high recruitment coincide with predominantly southwesterly winds and weak upwelling in spring and summer, which lead to an enhanced northward coastal current during the larval drift period. Also in most peak recruitment years, low-salinity anomalies are observed to propagate northward during the spring and summer. It is suggested that consistent southwesterly (downwelling) winds and propagating low-salinity anomalies, both leading to an enhanced northward transport of larvae, are important factors for elevated recruitment. At the same time, these conditions stabilize the coastal waters, possibly leading to enhanced production and improved feeding potential along the drift route to Barents Sea. Further studies on the drivers of early life history mortality can now be undertaken with a better understanding of the physical conditions that prevail during years when elevated recruitment occurs in this herring stock.

## Introduction

The recruitment of Norwegian Spring Spawning herring (NSSH) *Clupea harengus* L. is highly dynamic both in regard to the number of recruits and distribution of recruits across nursery areas [1–4]. The fjords along the Norwegian coast are important nursery grounds for NSSH, annually providing recruits to the stock but generally it is the juveniles that reside in the Barents Sea nursery that make up the majority of very strong year-classes [2, 5]. The size of the NSSH stock has been influenced by fishing pressure and shown to vary in phase with the temperature of the Atlantic Water throughout the 20^th^ century [6]. The long lasting lack of recovery during the 1970s was thought to be due to low spawning stock size, but also coincided with a period of relatively low water temperatures [6]. A direct causal link between temperature and recruitment or productivity has, however, not been established. Evidence for the linkage might prove challenging since temperature can act as a proxy for a number of other processes such as advection of water-masses or prey organisms, predator pressure, etc. [7]. Adding to this, studies of the North Sea herring indicate that the intertwining of environmental forcing, ecosystem state and other factors result in a complex relationship which affects survival in young herring [8].

**Fig 1:**
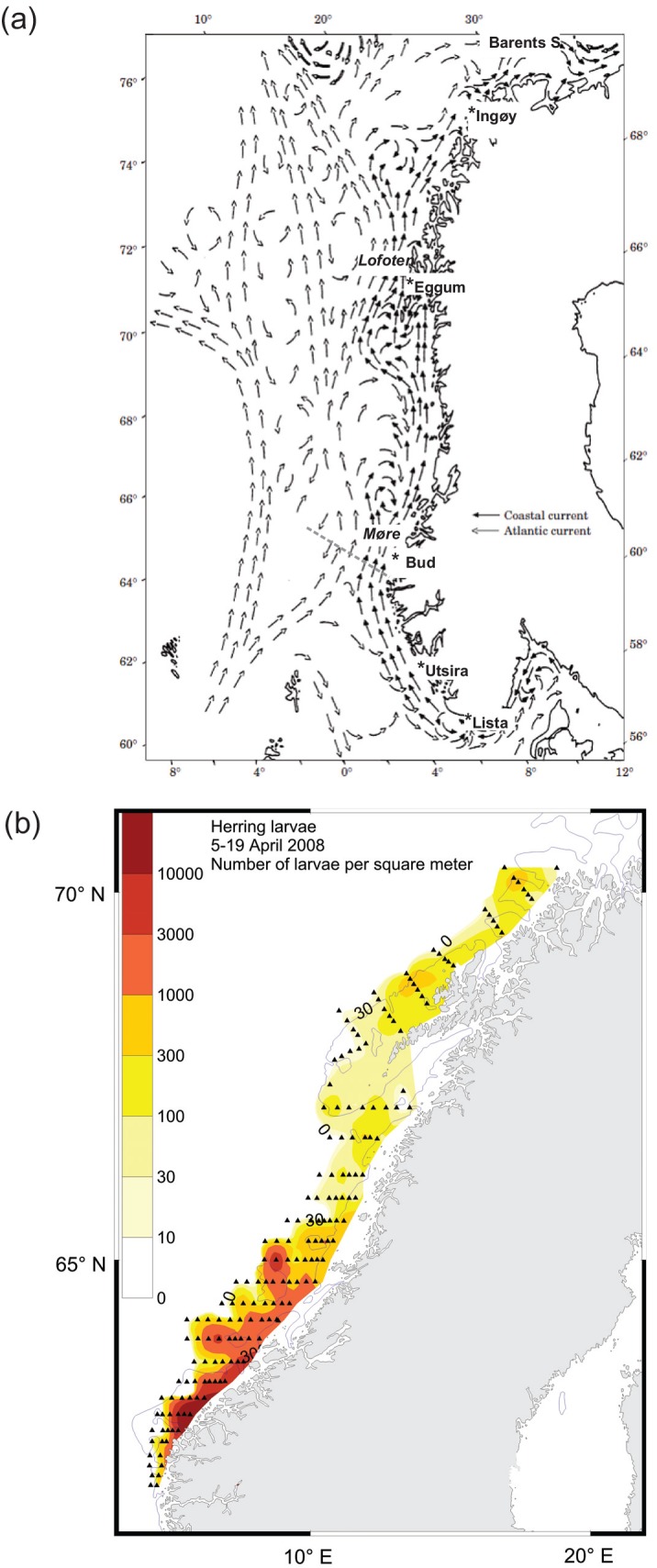
a) include a map of the study area showing the Norwegian Coastal Current and the Norwegian Atlantic Current, and the location of the fixed hydrographic stations [53], and b) distribution of herring larvae after hatching. b) A “normal” year 2008 is presented here to illustrate the distribution of herring larvae post hatch in the Norwegian Coastal Current [46]. Triangles represent larvae sampling stations. Dashed grey line indicates the Svinøy section.

A number of hypotheses have been suggested to explain recruitment variability in NSSH. Some have focused on larval feeding conditions [9] invoking the Critical Period hypothesis [1, 10] and the Match/Mismatch hypothesis formalized by Cushing [11, 12]. Others have focused on egg predation on the spawning grounds [13], transport mechanisms [14] or processes during the juvenile stages in the Barents Sea [15, 16] Tentative links have been made in the past between transport or retention mechanisms and survival of herring in the North Sea [17–19]. De Barros et al. [20] have shown that even if large numbers of NSSH larvae/juveniles arrive in the Barents Sea nursery grounds, the juvenile mortality rate can be so high that the cohort abundances are very low by the time they recruit to the adult population in the Norwegian Sea. However, this contradicts Sætre et al. [21] who stated that the year-class strength of NSSH is determined during the larval drift period along the coast. A number of studies, including Hjort [1], have emphasized the importance of drift mechanisms and the combination of biological and physical processes. Dragesund [2] noted that a widespread distribution of spawning sites, a long duration of the spawning period, and rapid northward dispersion of the larvae from the spawning grounds were advantageous for recruitment. The northward dispersion hypothesis has been supported by model simulations suggesting that early hatching will result in a rapid transport, which has been hypothesized as an important factor for recruitment [14, 22]. In contrast to this, several authors [21, 23–27] have suggested that retention of the larvae on coastal banks close to the spawning areas and wind-induced turbulence were important factors for larval survival due to improved feeding conditions. However, NSSH during the early life stages are transported over long distances (sometimes more than 1000 km) in the Norwegian Coastal Current (NCC) from the spawning grounds on coastal banks to the nursery areas (Fig 1). Thus, the environmental conditions in the NCC can be expected to influence survival during the northward transport phase and could play an important part in determining the resulting year class strength.

The hydrographic conditions and strength of the NCC are mainly determined by the freshwater input from the Baltic and Norwegian rivers and wind driven Ekman transports [28]. The strength of the NCC is mainly determined by the density contrast between the low-density coastal and the high-density Atlantic waters. Due to mixing, this density contrast decreases northward [28]. In the spring and summer, which coincides with the presence of early life stages of NSSH, irregular periods of upwelling-favorable northeasterly winds become frequent [29]. Increasing frequency and persistence of such events are expected to reduce the strength of the NCC [30] and hence reduce the northward transport rate of NSSH larvae.

The main purpose of this study is to investigate the inter-annual variability in the wind field and hydrography in the Norwegian coastal waters during the time when NSSH larvae are being transported northward from their spawning to nursery grounds. From the whole suite of hydrographic conditions that have occurred during the larval drift phase between 1948 and the present we identify what different hydrographic conditions prevailed specifically in years with high recruitment and elevated survival. The overall objective is thus to characterize the physical environmental conditions that coincide with elevated herring recruitment and provide an indicator of when elevated recruitment is likely to occur.

## Results

**Fig 2:**
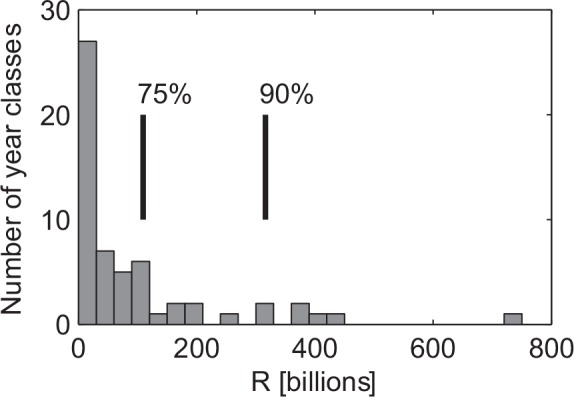
Histogram of the Norwegian Spring Spawning herring (*Clupea harengus* L.) recruitment during the period 1935–2010. The vertical lines are the 75_th%ile_ and the 90_th%ile_.

**Fig 3:**
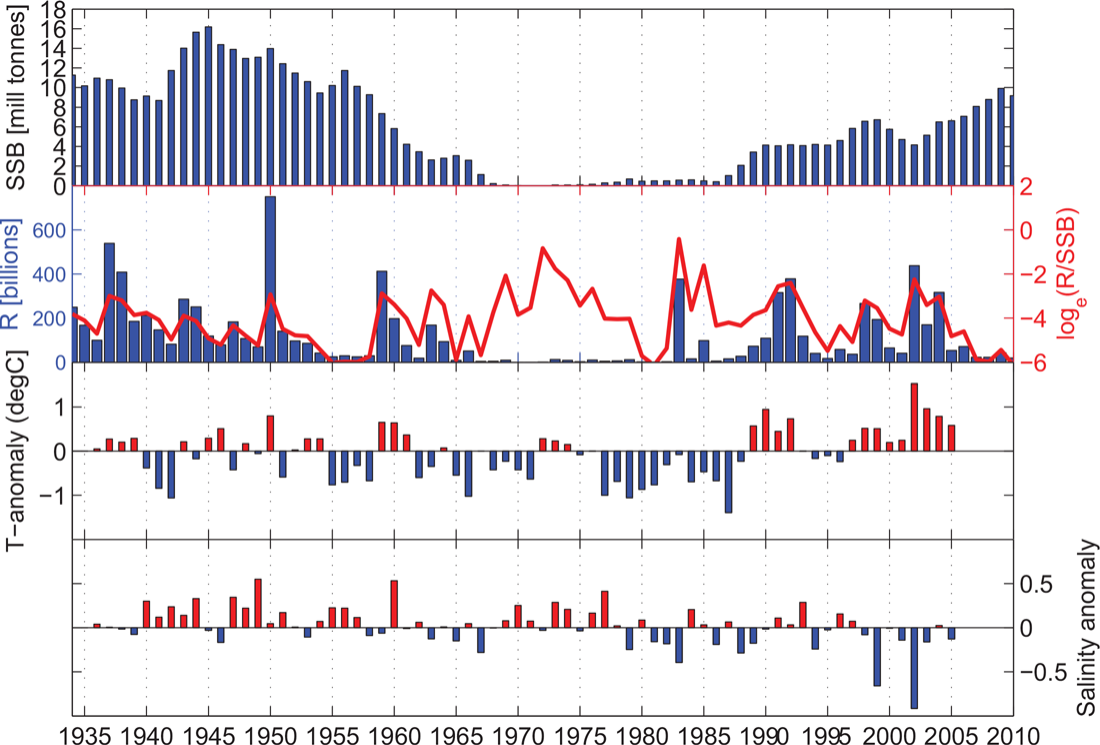
The development of the Norwegian Spring Spawning Herring stock showing a) the spawning stock biomass and b) the recruitment variability (both in absolute values and relative survival levels, given as Log (Recruit-per-Spawning Stock Biomass), compared to the hydrographic anomalies in the coastal waters at the Norwegian Coast; c) temperature and d) salinity. The Hydrographic anomalies are taken as the mean of the standardized values (subtracted mean and divided by standard deviation) at the fixed stations Utsira, Bud, Eggum and Ingøy in the upper [0–30] m.

The few very large recruitment years of the NSSH (Fig 2) suggest that the stock dynamics is essentially driven by these very infrequent episodic events (see Material and Methods section for details on the data). The long-term variability in the NSSH stock is prominent in the time series, with the decreasing stock during the 1960s, a collapsed state in the 1970s, and the recovery of the stock in the late 1980s (Fig 3A). Using a combined ranking of absolute numbers of recruits (R) and the survival rate from spawning stock biomass (SSB) to recruitment (R/SSB) the years with the top 10%, in rank order from highest to lowest are; 1983, 2002, 1950, 1992 1937, 1959, 1991 and 1938 (see Material and Methods for details). Many of the years with high recruitment and relative survival (Fig 3B) coincided with positive temperature anomalies (e.g. 1937, 1938, 1950, 1959, 1991, 1992 and 2002; Fig 3C). In regard to salinity, these peak recruitment years were close to the overall mean (1937, 1938, 1950, 1959, 1991 and 1992) or a strong negative anomaly (2002) (Fig 3D). Interestingly, the strong 1983 year class occurred during a period of very low spawning stock biomass, a moderately weak negative temperature anomaly but a relatively strong negative salinity anomaly. Using the method of [31] to estimate the number of effective samples (n_eff_) based on the autocorrelation in the series, we found a negative correlation between T and S (r = -0.31, p = 0.03, n_eff_ = 48), but interestingly, the negative correlation between their first derivatives were markedly higher (r = -0.46; p = 0.002, n_eff_ = 44). This negative correlation at year to year time scales could not be explained by the generally positive correlation between temperature and salinity in the inflow properties of the Atlantic water [32], but suggested the importance of local (regional) forcing.

**Fig 4:**
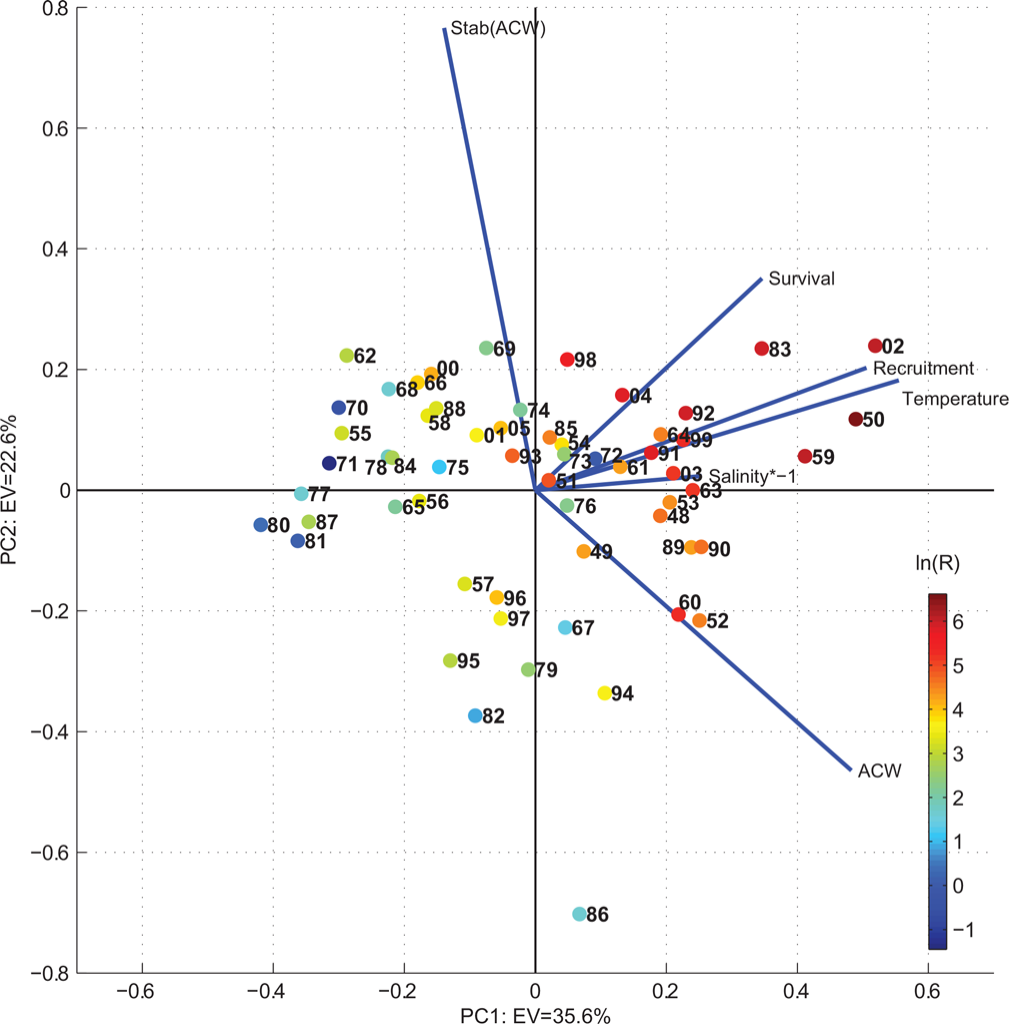
Scatter plot of the two leading principal components explaining respectively 35.6% (EOF1) and 22.6% (EOF2) of the total variance. The blue lines show the magnitude and sign of each variable's contribution to the first two principal components. Coloured points show the log_e_(Recruitment in billions). All variables are given equal weight by standardizing, i.e. subtracted mean and divided by its standard deviation prior to the analysis. The principal components are estimated using the matlab routine: *svd*.*m*

To resolve the covariance between the NSSH recruitment and the environmental conditions a principal component analysis (PCA) was performed (see Material and Methods section for details of the analysis). A scatter plot of the principal components 1 versus 2 showed that the above defined peak years of elevated recruitment grouped in the upper right quadrant, that is when the conditions were warmer and fresher than average and the along-coast wind (ACW—defined positive with the coast to the right) were stronger than average (Fig 4). The additional contribution from EOF 2 had a positive effect on the NSSH recruitment with increasing stability of the ACW (upper right quadrant in Fig 4).

The PCA analysis was based on mean values for the environmental parameters from March to August. Since the conditions in the Coastal Waters could change rapidly, there was interest in investigating how the hydrographic conditions change through the spring-summer period during these peak recruitment years in comparison to less successful years. We investigated these periods with respect to hydrographic variability captured at fixed stations (see Material and Methods part) during the drift period of the NSSH offspring. From south to north, averaged over the period March to August, the salinity increased and the temperature decreased (S1 Fig), and both contributed to weaker stratification. We focus on salinity since this represents the most robust measure of the coastal waters’ status, in comparison to temperature difference between the Coastal and Atlantic waters that varies and even changes sign over the year.

**Fig 5:**
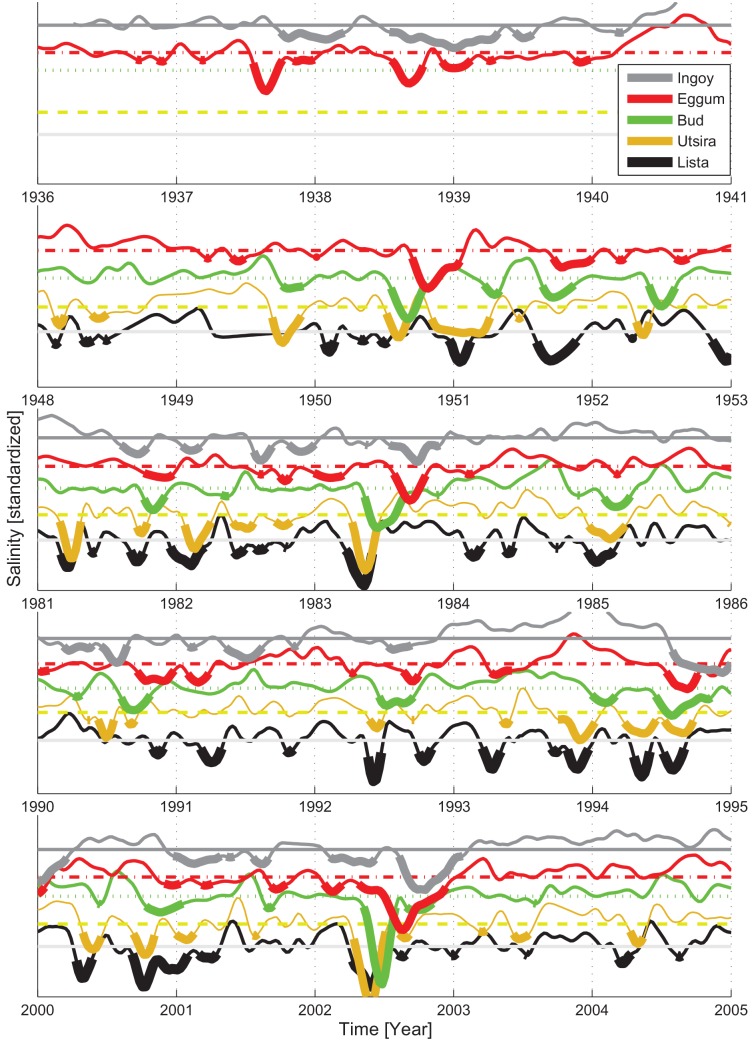
Hydrographic condition during five year intervals centered at the peak NSSH recruitment years 1937 and 1938, 1950, 1983, 1991 and 1992, and 2002. The series represent the upper, [0–30] m mean salinity from the fixed hydrographic stations. The mean annual cycle at each station and depth is removed, and the data are standardized i.e. deviation from mean and divided by the standard deviation. For visualization values < -.25 standard deviation are shown by thick lines.

In the following de-seasoned and standardized monthly-smoothed upper 30 m time-series of salinity were used to resolve spatio-temporal variability in physical conditions along the coast centered at the peak recruitment years; 1937 and 1938, 1950, 1983, 1991 and 1992, and 2002 (Fig 5). Note that data were sparse in 1959 (only a few stations) and this year was therefore not explored further in terms of hydrography. What appeared as a common feature in those years was a fresh water anomaly during spring and summer which propagated northward along the coast. This is most evident in the two highest ranked NSSH recruitment years, 1983 and 2002, where those anomalies originated in the south at Lista during spring and coincided with the drift of NSSH juveniles along the west coast, before entering the Barents Sea (station Ingøy) in the late summer. Also, during 1950 there was a clear fresh water signal, but this was less defined at the southernmost station and appeared a few months later in the year compared to 1983 and 2002. In 1992 there was a similar fresh water event, while in 1991 such event was only clear in the south (Lista) in spring and in the north (Svinøy) in early fall. In 1937 and 1938 we lack data for the three southern stations, but the fresh water anomalies in the late summer at Eggum and Ingøy are consistent with the other peak years. Considering adjacent years, only in 1994 a clear fresh water pulses progressed along the coast during the summer period, but these were different from the peak years when the stability of the ACW was anomalously low (see Fig 4, lower right quadrant). Further, considering the entire hydrographic data record from the fixed stations starting in 1936, such years when fresh water progressed over a large part of the coast during summer were rare, but they all (except 1994) coincided with elevated recruitment (not shown).

**Fig 6:**
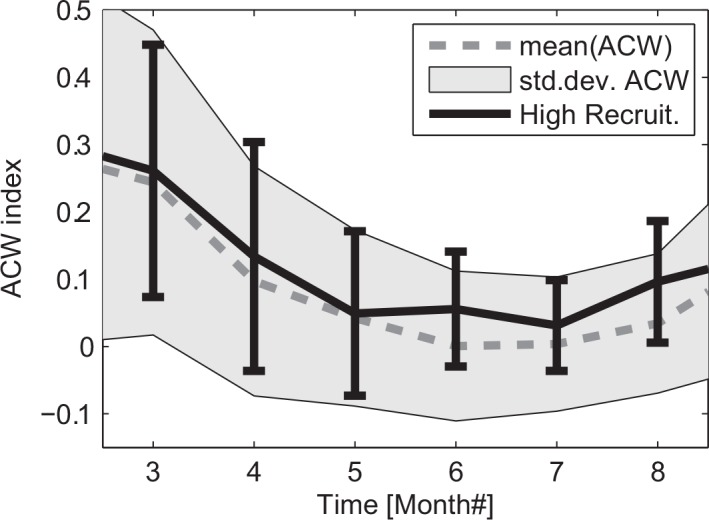
Indices for the Along Coast Wind component averaged between 64 to 68° N. The annual mean (dashed line) with corresponding standard deviation (gray area) are shown for the period 1948–2012. The similar values for the Along Coast Wind but limited to selected high NSSH recruitment years 1950, 1959, 1983 and 2002 are included with mean (solid line) and standard deviation (vertical bars). Note that the standard deviations are estimated based on weekly mean data.

The along-coast wind component provides the strongest impact on coastal systems as down-/upwelling occurs with the coast on the right/left of the downwind direction. The relatively large changes in the hydrography on inter-annual (Fig 3) and seasonal (Fig 5) time scale, suggested variable forcing connected to the wind field as a likely cause of the observed hydrographic changes. The average wind condition during the summer was characterized by positive ACW that became very weak during summer but was highly variable (Fig 6). Compared to this, during the high NSSH recruitment years the ACW were consistently stronger and with a lower variance. In particular, during the period June to August the ACW had a positive anomaly, with less variance compared to the long-term mean.

## Discussion

The results presented here show that strong year classes of Norwegian spring spawning herring during the period 1935–2009 occurred during years when low-salinity anomalies appeared and progressed from south to north during the spring-summer. These resulted from strong southeasterly winds and weak upwelling which lead to a consistent positive coastal current during the larval drift period. Also, in these years there was additional freshwater off the southern coast of Norway. This could support Dragesund’s [2] hypothesis that larval recruitment is high in years with a rapid northward dispersion into the Barents Sea nursery area and could coincide with a reduced overlap with predators along the coast [14, 22]. Results from particle tracking models documenting the rate of northward dispersion of larvae from the main spawning grounds off western Norway in 1989–2008 [22] lent support to the results of the present study; i.e. the largest year classes in this period were found in years with modeled positive temperature anomalies in combination with a rapid drift. The rapid drift shown in models [22] and field studies [14], is in contrast to the suggestion of a larval retention or ‘stationarity’ over the first two to three months of larvae after hatching put forward by Sinclair and Powers [27]. Further modeling work has also demonstrated that the southern spawning and subsequent northward dispersion generally resulted in a high spatio-temporal overlap of herring larvae with spring phytoplankton blooms developing along the larval drift route to the Barents Sea [33]. This indicated that rapid dispersion also may have been beneficial with regard to feeding success.

Variability in year class strength in fish populations could arise for a number of different reasons. Hjort [1] highlighted the inter-annual variability and raised a number of important hypotheses concerning their causes ranging from variable survival to advective losses of early life history stages. In regard to herring populations in particular, there is a considerable body of research on a number of stocks, however, each stock, whilst being the same species has a different ecology concerning the trajectory for life-history closure [34]. In contrast to the NSSH population, the NSAS herring population is comprised of a number of sub-stocks or components [35]. The question on what determines the year-class strength and whether different spawning components are influenced by the same process remain elusive [36]. Petitgas et al. [37] found that the different herring stock components in the North Sea experience different environmental forcing. The different coastal orientation of the North Sea means that atmospheric scale variability would project very differently onto regions of the various NSAS herring stocks.

Due to the relative constant orientation of the mid-Norway coastline combined with the large spatial scale of atmospheric forcing, conditions shift from favorable to unfavorable and *vice versa* over the entire region occupied by the early life history stages of NSSH. This very probably could explain why “one” environmental driver, e.g. specific wind condition or hydrographic conditions as explored here, could have a prominent effect on the NSSH population throughout periods of shifting climate from the warm 1930s, the cold 1960-70s, and the recent warm period, and accompanying changes in the size of the NSSH stock. Here we attempted to minimize density-dependent effects on herring (e.g. [38]) by considering time windows of five years centered around the peak recruitment years. The high recruitment in 1983 was exceptional since it developed despite a relatively small spawning stock (around 5x10^5^ tonnes) [39], and this year-class was considered to be exceptionally strong even as juveniles in August/September 1983 [40]. It was therefore of interest to characterize the conditions during 1983, as a basis for discussions on relationships between environmental conditions and recruitment during other years. The conditions during 1983 were characterized by a prominent freshwater anomaly that propagated northward along the entire Norwegian Coast (Fig 5). The timing corresponded roughly to the estimated drift of herring larvae, and this coincided with anomalously stable positive ACW (Figs 4 and 6).

There was a robust correspondence in that positive ACW, i.e. downwelling winds, were associated with higher NSSH recruitment (EOF1). Also, the analysis suggested that increased stability of the ACW (defined in Material and Method) coincide with increased recruitment under positive ACW conditions (Fig 4, upper right quadrant). Since the wind field and persistence of propagating freshwater anomalies were intimately linked we could not easily separate their individual contribution to NSSH recruitment. However, we can state that all the major propagating fresh water anomalies during summer coincided with increased NSSH recruitment. The high recruitment during 1950 was particularly interesting because the ACW was comparable to 1983, but the freshwater anomaly occurred some months later in the year. This could suggest that the stability of the water column through the early life history period, which was weaker in the upper layers in 1950 compared to e.g. 1983 and 2002, was not a crucial factor. However, it is also likely that the large stock in 1950 covering a larger spatio-temporal spawning is more robust to environmental perturbations and/or physio-biological factors, and thus sub-optimal conditions might have still been sufficient to have resulted in a high recruitment.

**Fig 7:**
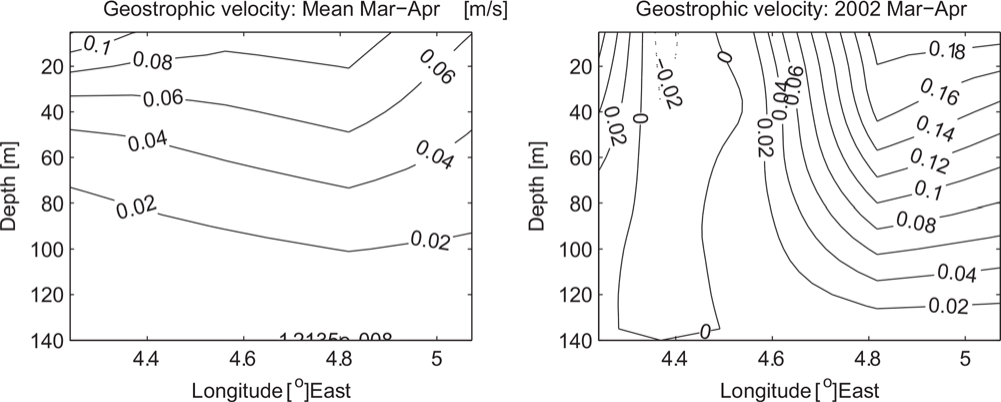
Baroclinic velocity field based on hydrographic sections over the continental shelf in the Svinøy section during the March and April 1986–2013. (Left) Mean velocities based on 47 sections and (right) velocity in 2002 which was a peak recruitment year based on two sections. The estimates are based on the assumption of a layer of zero velocity at 140 m which is nearly the depth of the shallowest station. Positive values means velocities northward and stations closest to the coast are to the right in the figures.

Through Ekman dynamics the ACW strongly affects environmental factors in the Coastal Waters of Norway. A positive ACW causes surface Ekman transport toward the coast and thus acts to maintain hydrographic anomalies and sea level to build toward the coast. This acts to increase the barotropic component of the Norwegian Coastal Current that is proportional to the ACW [30], and also the baroclinic component connected to horizontal density gradients. Based on 47 repeated hydrographic sections, obtained from 1986 to 2013 in the Svinøy section crossing the NCC at about 63 °N, we compare the baroclinic velocity field for the mean and the peak NSSH recruitment year 2002 (Fig 7). It should be noted that all section are obtained during March and April to coincide spatio-temporally with the juvenile NSSH stages. Consistent with previous discussion, the coastal current in 2002 is confined to the coast with a relatively large velocity shear toward the surface reaching ~18 cm/s. In comparison the mean velocity field is characterized by a weaker and offshore displaced NCC. Furthermore, based on the same data the core NCC velocity, defined as the mean velocity of the upper 30 m within 20 km from the coast, is at maximum during 2002 over to the period 1986–2013 where data are available (not shown). A persistent ACW would thus increase the probability of successful transport to the Barents Sea nursery areas. A similar mechanism has been proposed for cod in the Gulf of Maine [41, 42] and anchovy in the southern Benguela Current [43]. In contrast, a negative ACW would result in offshore Ekman transport, upwelling at the coast and decreasing sea level, both contributing to a weaker NCC. In addition, offshore surface Ekman transport would tend to transport herring larvae westward of the main northward flow into the Barents Sea, and possibly to less favorable conditions in terms of food availability and predators as e.g. for mackerel (*Scomber scombrus)* [44].

Despite the generally positive correlation between the ACW and the NSSH recruitment (e.g. Fig 4, PCA1), these data indicated that there was an “optimal environmental window” [45]; here it appeared that above a threshold ACW the herring larvae became advected into the fjords along the Norwegian coast. In 1994 there was a high ACW but low NSSH recruitment. Simulation studies showed that the larvae had a pronounced inshore drift pattern increasing the probability of being transported into one of many fjords along the coast [46]. The fjords are thought to be important nursery grounds for smaller year-classes, but the strong year-classes of NSSH originate mainly from nursery areas in the Barents Sea [2, 5].

Toresen and Østvedt [6] have shown that, over a multidecadal scale, the size of the NSSH stock was positively correlated with the Kola transect sea temperatures. Slotte and Fiksen [47] and [48] have shown a similar relationship with temperatures from the same fixed stations used in this study. Also, in the present study, the peak recruitment years coincided with relative maxima in the temperature. However, in 1983, which we hypothesized as a year with “optimal” conditions for recruitment, the temperature in the coastal water was still below the long-term mean. Notably, changes in the along-coast wind component, in particular when going from down- to upwelling winds, immediately leads to upwelling of cold water. It was therefore probably not the direct effect of temperature on the recruitment, but more that high temperature was connected to anomalous weak upwelling. It should also be noted that the spawning stock was extremely small in the years around 1983 and the distribution and migration patterns of the stock were more coastal than oceanic [49]. We do not address potential effect of spawning variability between years and spawning grounds. However, in contrast to the pronounced, ‘spiked’ pattern seen in the recruitment time series, the larvae data indicate a gradual annual change in the peak hatching dates.

**Fig 8:**
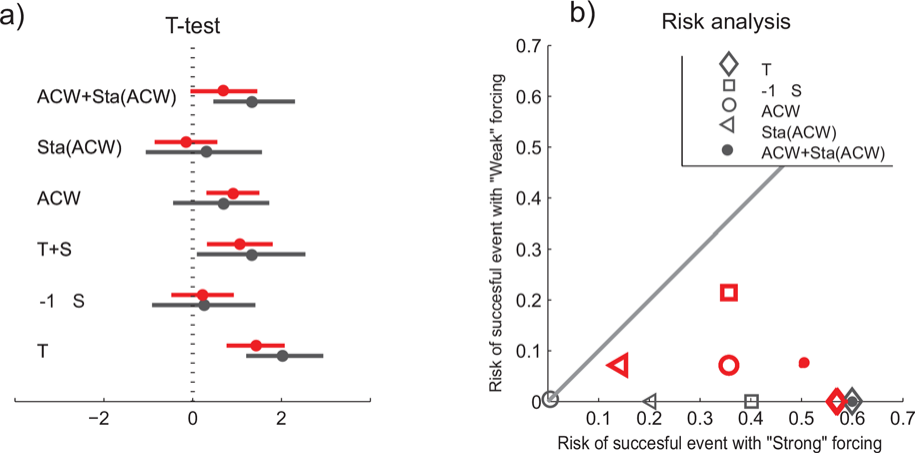
The recruitment success during years of “strong” compared to years of “weak” environmental forcing, i.e. [T, S, ACW, Stability(ACW), ACW+Stability(ACW)] > 90 _th%ile_ compared to [T, S …] < 10 _th%ile_ (black), and similarly for [T, S …] > 75_th%ile_ compared to [T, S …] < 25_th%ile_ (red). In a) results of a t-test including the mean and the 95% confidence interval, and b) a risk analysis to compare the chance of recruitment success during years of “strong” and “weak” environmental forcing. In a) positive values and in b) points below the line 1:1, corresponding to equal risk, indicate that the chance of successful recruitment is higher with stronger environmental forcing, and vise versa. In this analysis all data are standardized, i.e. for the respective series the mean is subtracted and then divided by the standard deviation. Recruitment is taken as log_e_(R), and Salinity (S) is multiplied by -1 in this analysis.

The ecological responses to extreme events have received little attention, relative to studies of trends and mean values [50]. The few very large recruitment years of the NSSH indicate the importance of such extreme years. Here we follow Mantzouni and MacKenzie [51] to investigate if the recruitment differs during years of “strong” and “weak” environmental forcing. A t-test indicates that for both the coastal temperature and the combined effect of velocity and stability of the ACW, the recruitment is significantly higher during “strong” compared to the “weak” forcing years selected based on the 75^th^ and the 90^th^ percentile (Fig 8A). Similarly when plotting the probabilities during years of “strong” versus years of “weak” forcing all points fall below the no-effect line, also indicating that the chance for successful recruitment is higher with the defined “strong” environmental forcing (Fig 8B). Thus, in terms of prediction, both the temperature and the additive effect of the ACW and its stability, through the spring and summer, could be useful indicators of the likelihood of successful recruitment.

In our study we combined the output from the Virtual Population Analysis (VPA) undertaken by Toresen and Østvedt [6] to assess the size of the Norwegian spring spawning herring population with the standard output from a recent ICES analysis [39]. Due to many uncertainties in the data during the period when the stock was collapsed, the current ICES Assessment Working Group does not report stock sizes prior to 1986. To extend the time series prior to 1986 it was necessary to combine data sets. We recognize that each of the methods used to generate the earlier and later time series gave different absolute levels of stock size and recruitment but where the time series did overlap very similar trends in stock and recruitment were apparent. As [52] pointed out, care must be taken with using any time series of recruitment but we felt that our combined data series were adequate for the present analyses. Probably the largest area for concern was in the estimate of survival (R/SSB), however, we avoided presenting or discussing this in absolute terms.

## Concluding Remarks

In summary, recruitment in NSSH is a complex process and there are likely to be many contributory factors to the formation of large year classes and/or enhanced survival rates through the early life history stages. Here, we showed that the common environmental conditions during the peak NSSH recruitment years were freshwater anomalies that progress from south to north early in the year along with persistent southwesterly winds. These conditions contributed to a rapid transport of larvae from the spawning grounds northward to nurseries in the Barents Sea. These conditions also contributed to stabilization of the water column, which in turn would affect primary production and food availability. To quantify these effects was beyond the scope of this study. However, further studies on the drivers of early life history mortality can now be undertaken with a better understanding of the physical conditions that prevail during years when elevated recruitment occurs in this herring stock.

## Material and Methods

### Physical data sources

Data from five fixed oceanographic stations [53] along the Norwegian coast (Fig 1) were used to analyze the variability in the Norwegian Coastal Current (NCC). The fixed station observations started between 1935 and 1946, with depth profiles generally being obtained twice per month (Table 1). Before the early 1960s, the standard depths were 1, 10, 25, 50, 75, 100, 125, 150, 200, 250, and 300 m, while later observations were taken at 20 and 30 m instead of 25 m. Measurements from these stations were taken with Nansen bottles equipped with reversing thermometers. From the 1990s measurements were made with mini Conductivity Temperature Depth (CTD) recorders (SAIV SD204 instrument). Calibration of the salinity measurements were made from Nansen water bottle samples. The accuracy of the data prior to the CTDs was approximately ± 0.03°C for temperature and ±0.03 for salinity. The accuracy of the CTDs given by the manufacture of the instruments is ± 0.01°C and ± 0.02 for salinity, and ± 0.02% of the range (500 dBar) for the pressure sensor.

**Table 1:** Key characteristics of the fixed oceanographic stations.

Station’s name	Latitude	Longitude	Bottom depth [m]	Sampling interval [per month]	Earliest observation
Ingøy	71°08’N	24°01 E	310 m	2.4	March 1936
Eggum	68°22.8’ N	13°38’ E	210 m	1.8	February 1935
Bud	62°56’ N	06°47’ E	268 m	2.2	February 1946
Utsira, outer	59°19’ N	04°44’ E	270 m	2.2	February 1942
Lista	58°05.1’ N	06°32’ E	310 m	2.2	January 1942

The atmospheric forcing data are taken from the NCEP/NCAR reanalysis [54] starting in 1948 and provided at 2.5° latitude and longitude spatial resolution and 6 hour time interval. From these data we calculate the along-coast component of the wind stress.

### Physical data indices

Prior to utilizing the hydrographic data the profiles were interpolated vertically to 1 m resolution, and then interpolated over time to give daily values for each depth. In order to extract the inter-annual hydrographic anomalies in the NCC we removed the seasonal cycle that otherwise would dominate the variability. Thus, from the observations we estimated the seasonal cycles at each station at each depth. These were then subtracted from the data to produce seasonally adjusted anomalies. The station-specific March to August [0–200 m] depth-mean variations are shown in S1 Fig. In addition, these mean profiles were subtracted stations-wise to give the March to August period anomalies during the peak years focusing on the upper 0–100 m (S2 Fig). The coastal temperature and salinity series, presented as the March to August upper [0–30] m overall mean at Utsira, Bud, Eggum and Ingøy, after subtracted mean and divided by standard deviation at the individual fixed stations, are illustrated in Fig 3. In Fig 5 station-wise monthly smoothed [0–30] m depth-mean salinity anomalies (divided by the standard deviation) are presented for selected years.

From the NCEP/NCAR data set we estimated the along-coast wind-stress (ACW) using the local orientation of the main coastline and take the average from 64 to 68°N. Then a one-week moving average filter was applied to these averaged data to produce the along-coast wind index (ACW-index). These weekly averages were used to estimate the monthly mean and monthly standard deviation of the ACW (Fig 6).

### Herring recruitment data

The time series of recruitment (numbers at age 0) of NSSH from 1988–2011 were obtained from results of analytical assessment of ICES [39]. The preceding years (1935–1987) were taken from [6], however this utilized a different analytical technique than used for later years and probably results in a change in absolute estimation of abundances. Any bias, however, will not affect the identification of good and poor year classes. For the period prior to 1988, Toresen and Østvedt [6] state that with the exception of years encompassing the Second World War (1939–1945), when the catch statistics and the sampling were probably of a poor quality, the data give a reliable indication of year-class strength.

The few very large recruitment years of the NSSH (Fig 2) suggest that the stock dynamics is essentially driven by these very infrequent episodic events. To define “peak” recruitment years the data are ranked with respect to absolute recruitment (R) and relative recruitment (survival) defined as R/SSB with SSB being the stock spawning biomass. We then sort the years with respect to the highest total ranking (lowest value) and define the 10% highest ranking as peak recruitment years. In descending order the peak years consists of the following years; 1983, 2002, 1950, 1992 1937, 1959, 1991 and 1938 (see S2 Table). The cutoff at 10% is somewhat arbitrary. However, the strategy here is to examine the highest ranked years in detail, and to investigate if identified environmental conditions also occur, but possible less clear, in other years with relatively high recruitment. A statistical analysis of the high recruitment years and the physical conditions was undertaken following the methods laid out in Mantzouni and MacKenzie [50]. Here the intention was to investigate if the recruitment differs during years of “strong” and “weak” environmental forcing. To examine the robustness of our analyses we also undertook the analyses both using the 90/10^th^ and 75/25^th^ percentiles of recruitment abundance.

### Principal component analysis

The variables entering the principal component analysis (e.g. [55]) were ln(R), ln(R/SSB), T-index, S-index, ACW component, stability of the ACW. Here R was the recruitment, SSB was the spawning stock biomass, ACW was the along coast wind, and the stability of the ACW was defined as -1*variance(ACW). Further, prior to the analysis the series were standardized, i.e. the mean was removed and then divided by standard deviation. For the analysis we had a complete data matrix over the period 1948 to 2005. To extract the principal components the Matlab routine *svd*.*m* was applied to the data. Only the two leading principal components containing in total 58.4% of the total variance were considered.

## Supporting Information

S1 Fig The mean hydrographic conditions during the period from March to August based on the fixed stations Lista, Utsira, Bud, Eggum and Ingøy. (EPS)

S2 Fig The March to August mean anomalous left) salinity and right) temperature for the high NSSH recruitment years 1937, 1938, 1950, 1959, 1983, 1992 and 2002 (see legend for color table). Starting in the south at Lista—upper figures, proceed northwards with Utsira, Bud, Eggum and ending in the north at Ingøy–lower figures.
(EPS)

S1 Table Norwegian Spring Spawning herring (Clupea harengus L.) recruitment data. (XLS)

S2 Table The Norwegian Spring Spawning Herring recruitment data; R is the absolute recruitment (in billions), R/SSB is a measure of survival. The data are sorted according to their total ranking with respect to recruitment (R) and survival (R/SSB). The 10% years with highest ranking are shown by grey.
(DOC)

## References

[1] Hjort J (1914) Fluctuations in the great fisheries of northern Europe viewed in light of biological research. Rapp P-v Réun Cons Perm Int Explor Mer. 20: 1–228.

[2] Dragesund O (1970) Factors influencing year-class strength of Norwegian spring spawning herring (*Clupea harengus* L.). Fisk Skrift Ser Havunders. 15: 381–450.

[3] Dragesund O (1971) Comparative analysis of year-class strength among fish stocks in the North Atlantic. Fisk Skrift Ser Havunders. 16: 49–64.

[4] Toresen R, Østvedt OJ (2002) Stock structure of Norwegian spring-spawning herring: historical background and recent apprehension. ICES Mar Sci Symp. 215: 532–542.

[5] Holst JC, Slotte A (1998) Effects of juvenile nursery on geographic spawning distribution in Norwegian spring-spawning herring (*Clupea harengus L*.). ICES J Mar Sci. 55: 987–996.

[6] Toresen R, Østvedt OJ (2000) Variations in abundance of Norwegian spring-spawning herring (*Clupea harengus* L.) throughout the 20th century and the influence of climatic fluctuations. Fish Fish. 1: 231–256.

[7] Sundby S (2000) Recruitment of Atlantic cod stocks in relation to temperature and advection of copepod populations, Sarsia. 85(4): 277–298.

[8] Hufnagl M, Peck MA, Nash RDM, Dickey-Collas M (2014) Unravelling the Gordian knot! Key processes impacting overwintering larval survival and growth: A North Sea herring case study. Prog Oceanogr. doi: 10.1016/j.pocean.2014.04.029

[9] Fossum P (1996) A study of first-feeding herring (*Clupea harengus L*.) larvae during the period 1985–1993. ICES J Mar Sci. 53: 51–59.

[10] Hjort J (1926) Fluctuations in the year classes of important food fishes. J Cons int Explor Mer. 1: 5–38.

[11] Cushing DH (1975) Marine Ecology and Fisheries. Cambridge University Press, Cambridge, 278 pp.

[12] Cushing DH (1990) Plankton production and year-class strength in fish populations: an update of the match/mismatch hypothesis. Adv Mar Biol. 26: 249–293.

[13] Toresen R (1991) Absorption of acoustic energy in dense herring schools studied by the attenuation in the bottom echo signal. Fish Res. 10: 317–327.

[14] Husebø Å, Stenevik EK, Slotte A, Fossum P, Salthaug A, Vikebø F, et al (2009) Effects of hatching time on year-class strength in Norwegian spring-spawning herring (*Clupea harengus*). ICES J. Mar Sci. 66: 1710–1717.

[15] De Barros P, Tirasin EM, Toresen R (1998) Relevance of cod (*Gadus morhua* L.) predation for inter-cohort variability in mortality of juvenile Norwegian spring spawning herring (*Clupea harengus* L.). ICES J. Mar Sci. 55: 454–466.

[16] Johansen GO, Bogstad B, Mehl S, Ulltang Ø (2004) Consumption of juvenile herring (*Clupea harengus*) by cod (*Gadus morhua*) in the Barents Sea: a new approach to estimating consumption in piscivorous fish. Can J Fish Aquat Sci. 61: 343–359.

[17] Bartsch J, Brander K, Heath M, Munk P, Richardson K, Svendsen E (1989) Modelling the advection of herring larvae in the North Sea. Nature. 340: 632–636.

[18] Nichols JH, Brander KM (1989) Herring larval studies in the west-central North Sea. Rapp P-v Réun Cons Int Explor Mer. 191: 160–168.

[19] Dickey-Collas M, Nash RDM, Brunel T, van Damme CJC, Marshall CT, Payne MR, et al (2010) Lessons learned from stock collapse and recovery of North Sea herring; a review. ICES J Mar Sci. 67: 1875–1886.

[20] De Barros P, Tirasin EM, Toresen R (1998) Relevance of cod (*Gadus morhua* L.) predation for inter-cohort variability in mortality of juvenile Norwegian spring spawning herring (*Clupea harengus* L.). ICES J Mar Sci. 55: 454–466.

[21] Sætre R, Toresen R, Anker-Nilssen T (2002a) Factors affecting the recruitment variability of the Norwegian spring-spawning herring (*Clupea harengus* L.). ICES J Mar Sci. 59: 725–736.

[22] Vikebø FB, Husebø Å, Slotte A, Stenevik EK, Lien VS (2010) Effect of hatching date, vertical distribution, and interannual variation in physical forcing on northward displacement and temperature conditions of Norwegian spring-spawning herring larvae. ICES J Mar Sci. 67: 1948–1956.

[23] Iles TD, Sinclair M (1982) Atlantic herring: stock discreteness and abundance. Science. 215:627–633. 17842372 10.1126/science.215.4533.627

[24] Sinclair M, Tremblay MJ (1984) Timing of spawning of Atlantic herring (*Clupea harengus harengus*) populations and the match–mismatch theory. Can J Fish Aquat Sci. 41: 1055–1065.

[25] Sinclair M (1988) Marine Populations, An Essay on Population Regulation and Speciation. Washington Sea Grant, Seattle.

[26] Sætre R, Toresen R, Søiland H, Fossum P (2002b) The Norwegian spring-spawning herring—spawning, larval drift and larval retention. Sarsia. 87(2): 167–178.

[27] Sinclair M, Power M (2015) The role of “larval retention” in life-cycle closure of Atlantic herring (*Clupea harengus*) populations. Fish Res. 172: 401–414.

[28] Helland-Hansen B, Nansen F (1909) The Norwegian Sea: Its physical oceanography based upon the Norwegian research 1900–1904. *In* Report on Norwegian Fisheries and Marine Investigations, 2(1), 390 pp.

[29] Iden KA (1997) Meteorologi–værforholdene på Norskekysten, in Den Norske Los, vol. I, pp. 129–146, Statens kartverk Sjøkartverket, Stavanger, Norway.

[30] Skagseth Ø, Drinkwater K, Terrile E (2011) Wind and buoyancy induced transport of the Norwegian Coastal Current in the Barents Sea. J Geophys Res. 116, C08007, 10.1029/2011JC006996

[31] Pyper BJ, Peterman RM (1998) Comparison of methods to account for autocorrelation in correlation analyses of fish data. Can J Fish Aquat Sci. 55(9): 2127–2140.

[32] Skagseth Ø, Furevik T, Ingvaldsen R, Loeng H, Mork KA, Orvik KA, et al (2008) Volume and heat transports to the Arctic via the Norwegian and Barents Seas, pp. 45–64. In Arctic-Subarctic Ocean Fluxes: Defining the role of the Northern Seas in Climate. Eds. Dickson R., Meincke J. and Rhines P., Springer Netherlands, 10.1007/978-1-4020-6774-7

[33] Vikebø FB, Korosov A, Stenevik EK, Husebø Å, Slotte A (2012) Spatio-temporal overlap of hatching in Norwegian spring-spawning herring and the spring phytoplankton bloom at available spawning substrata. ICES J Mar Sci. 69: 1298–1302.

[34] Petitgas P, Rijnsdorp AD, Dickey-Collas M, Engelhard GH, Peck MA, Pinnegar JK, et al (2013) Impacts of climate change on the complex life cycles of fish. Fish Oceanogr. 22(2): 121–139.

[35] Payne MR (2010) Mind the gaps: a state-space model for analysing the dynamics of North Sea herring spawning components. ICES J Mar Sci. 67: 1939–1947.

[36] Cushing DH, Bridger JP (1966) The stock of herring in the North Sea and changes due to fishing. Fish Invest, Lond Ser 2, 25: (1) 123 pp.

[37] Petitgas P, Huret M, Léger F, Peck MA, Dickey-Collas M, Rijnsdorp AD (2009) Patterns and schedules in hindcasted environments and fish life cycles. ICES Document CM 2009/E:25. 12 pp.

[38] Nash RDM, Dickey-Collas M, Kell LT (2009) Stock and recruitment in the North Sea hering (*Clupea harengus*); compensation and depensation in the population dynamics. Fish Res. 95: 88–97.

[39] ICES (2013) Report of the Working Group on Widely Distributed Stocks (WGWIDE). ICES CM 2012/ACOM:15. 645 pp.

[40] Røttingen I (1989) The 1983 year class of Norwegian spring spawning herring as juveniles and recruit spawners. pp. 165–203. *In*: Monstad, T. (Ed.) Biology and Fisheries of the Norwegian Spring-Spawning Herring and Blue Whiting in the Northeast Atlantic. Proceedings of the Fourth Soviet-Norwegian Symposium, 12–16 June 1989. Bergen, Norway: Institute of Marine Research, 385 pp.

[41] Runge JA, Kovach AI, Churchill JH (2010) Understanding climate impacts on recruitment and spatial dynamics of Atlantic cod in the Gulf of Maine: Integration of observations and modeling. Prog Oceanogr. 87: 251–263.

[42] Churchill JH, Runge J, Chen C (2011) Processes controlling retention of spring-spawned Atlantic cod (*Gadus morhua*) in the western Gulf of Maine and their relationship to an index of recruitment success. Fish Oceanogr. 20(1): 32–46.

[43] Parada C, Mullon C, Roy C, Freon P, Hutchings L, van der Lingen CD (2008) Does vertical migratory behaviour retain fish larvae onshore in upwelling ecosystems? A modelling study of anchovy in the southern Benguela. African J Mar Sci. 30(3): 437–452.

[44] Skaret G, Bachiller E, Langøy H, Stenevik EK (2015) Mackerel predation on herring larvae during summer feeding in the Norwegian Sea. ICES J Mar Sci. 10.1093/icesjms/fsv087

[45] Cury P, Roy C (1989) Optimal environmental window and pelagic fish recruitment success in upwelling areas. Can J Fish Aquat Sci. 46: 670–680.

[46] Stenevik EK, Nash RDM, Vikebø F, Fossum P, Bakkeplass K (2012) The effects of survey design and circulation pattern on the perceived abundance of herring larvae: a case study for Norwegian spring spawning herring (*Clupea harengus*). Fish Oceanogr. 21(5): 363–372.

[47] Slotte A, Fiksen Ø (2000) State-dependent spawning migration in Norwegian spring-spawning herring. J Fish Biol. 56: 138–162.

[48] Fiksen Ø, Slotte A (2002) Stock-environment recruitment models for Norwegian spring spawning herring (*Clupea harengus*). Can J Fish Aquat Sci. 59: 211–217.

[49] Holst JC, Røttingen I, Melle W (2004) The herring Pp. 203–226. In: Skjoldal H.R., Sætre R., Færnö A., Misund O.A. and Røttingen I. (eds.) The Norwegian Ecosystem. Tapir Academic Press, Trondheim, Norway.

[50] Easterling DR, Meehl GA, Parmesan C, Changnon SA, Karl TR, Mearns LO (2000) Climate extremes: observations, modelling, and impacts. Science. 289: 2068–2074. 11000103 10.1126/science.289.5487.2068

[51] Mantzouni I, MacKenzie BR (2010) Productivity responses of a widespread marine piscivore, *Gadus morhua*, to oceanic thermal extremes and trends. Proc R Soc B, 10.1098/rspb2009.1906, PMC2871868 20147332

[52] Dickey-Collas M, Payne MR, Trenkel VM, Nash RDM (2014) Hazard warning: model misuse ahead. ICES J Mar Sci. 71: 2300–2306.

[53] Aure J, Østensen Ø (1993) Hydrographic normals and long-term variations in Norwegian coastal waters. Fisk Hav. 6, pp. 7.

[54] Kalnay E, Kanamitsu M, Kistler R, Collins W, Deaven D, Gandin L, Iredell M, et al (1996) The NCEP/NCAR 40-year reanalysis project. Bull Am Met Soc. 77: 437–471.

[55] Preisendorfer RW (1988) Principal component analysis in meteorology and oceanography In: Development in Atmospheric Science, Vol. 17 Elsevier, Amsterdam, 425 pp.

