# Supplementary material for: Characteristics of the Norwegian Coastal Current during Years with High Recruitment of Norwegian Spring Spawning Herring (Clupea harengus L.)
Source: PLoS One. 2015 Dec 4;10(12):e0144117. doi: 10.1371/journal.pone.0144117 (PMC4670105; doi:10.1371/journal.pone.0144117)
Supplement: S2 Table — The data are sorted according to their total ranking with respect to recruitment (R) and survival (R/SSB). The 10% years with highest ranking are shown by grey. (DOC) [file pone.0144117.s004.doc]

S2 Table. The Norwegian Spring Spawning Herring recruitment data; R is the absolute recruitment (in billions), R/SSB is a measure of survival. The data are sorted according to their total ranking with respect to recruitment (R) and survival ( R/SSB). The 10% years with highest ranking are shown by grey.

| Year | R | R/SSB | Rank(R) | Rank(R/SSB)) | Sum Rank | Year | R | R/SSB | Rank(R) | Rank(R/SSB)) | Sum Rank | |
| --- | --- | --- | --- | --- | --- | --- | --- | --- | --- | --- | --- | --- |
| **1983** | **377** | **0,66** | **7** | **1** | **8** | 1984 | 16 | 0,027 | 57 | 25 | 82 |  |
| **2002** | **438** | **0,106** | **3** | **6** | **9** | 1996 | 59 | 0,013 | 39 | 45 | 84 |  |
| **1950** | **751** | **0,054** | **1** | **13** | **14** | 2000 | 66 | 0,012 | 38 | 46 | 84 |  |
| **1992** | **379** | **0,091** | **6** | **8** | **14** | 2006 | 72 | 0,011 | 36 | 48 | 84 |  |
| **1937** | **539** | **0,05** | **2** | **14** | **16** | 1953 | 86 | 0,009 | 31 | 54 | 85 |  |
| **1959** | **413** | **0,057** | **4** | **12** | **16** | 1942 | 82 | 0,007 | 32 | 57 | 89 |  |
| **1991** | **317** | **0,078** | **9** | **9** | **18** | 1975 | 3 | 0,033 | 70 | 21 | 91 |  |
| **1938** | **409** | **0,042** | **5** | **16** | **21** | 1946 | 80 | 0,006 | 33 | 59 | 92 |  |
| 2004 | 317 | 0,049 | 8 | 15 | 23 | 1988 | 28 | 0,014 | 49 | 44 | 93 |  |
| 1998 | 267 | 0,041 | 11 | 17 | 28 | 1968 | 6 | 0,024 | 67 | 27 | 94 |  |
| 1963 | 169 | 0,065 | 19 | 11 | 30 | 1994 | 41 | 0,01 | 45 | 49 | 94 |  |
| 1985 | 99 | 0,2 | 28 | 3 | 31 | 2001 | 42 | 0,009 | 44 | 51 | 95 |  |
| 1960 | 198 | 0,034 | 14 | 18 | 32 | 2005 | 54 | 0,009 | 40 | 55 | 95 |  |
| 2003 | 171 | 0,034 | 18 | 20 | 38 | 1979 | 13 | 0,018 | 60 | 36 | 96 |  |
| 1999 | 195 | 0,029 | 15 | 24 | 39 | 1949 | 71 | 0,006 | 37 | 61 | 98 |  |
| 1940 | 213 | 0,024 | 13 | 28 | 41 | 1971 | 1 | 0,03 | 76 | 22 | 98 |  |
| 1943 | 286 | 0,021 | 10 | 32 | 42 | 1987 | 16 | 0,015 | 58 | 41 | 99 |  |
| 1939 | 186 | 0,022 | 16 | 31 | 47 | 1978 | 7 | 0,018 | 65 | 37 | 102 |  |
| 1993 | 119 | 0,03 | 24 | 23 | 47 | 1977 | 6 | 0,018 | 68 | 35 | 103 |  |
| 1964 | 94 | 0,034 | 30 | 19 | 49 | 1970 | 1 | 0,022 | 75 | 29 | 104 |  |
| 1990 | 110 | 0,027 | 25 | 26 | 51 | 1997 | 37 | 0,007 | 46 | 58 | 104 |  |
| 1944 | 251 | 0,017 | 12 | 40 | 52 | 1954 | 43 | 0,005 | 43 | 63 | 106 |  |
| 1935 | 168 | 0,017 | 20 | 39 | 59 | 2009 | 43 | 0,005 | 42 | 64 | 106 |  |
| 1941 | 148 | 0,017 | 21 | 38 | 59 | 1986 | 6 | 0,014 | 66 | 43 | 109 |  |
| 1947 | 184 | 0,014 | 17 | 42 | 59 | 1958 | 30 | 0,004 | 47 | 68 | 115 |  |
| 1973 | 13 | 0,172 | 59 | 4 | 63 | 1962 | 20 | 0,006 | 55 | 60 | 115 |  |
| 1989 | 73 | 0,022 | 35 | 30 | 65 | 1956 | 30 | 0,003 | 48 | 72 | 120 |  |
| 1969 | 10 | 0,126 | 62 | 5 | 67 | 1995 | 18 | 0,005 | 56 | 65 | 121 |  |
| 1961 | 77 | 0,018 | 34 | 34 | 68 | 2007 | 23 | 0,003 | 52 | 69 | 121 |  |
| 1951 | 141 | 0,012 | 22 | 47 | 69 | 1957 | 26 | 0,003 | 50 | 73 | 123 |  |
| 1974 | 9 | 0,102 | 63 | 7 | 70 | 2008 | 23 | 0,003 | 53 | 71 | 124 |  |
| 1976 | 11 | 0,069 | 61 | 10 | 71 | 1955 | 25 | 0,003 | 51 | 74 | 125 |  |
| 1966 | 52 | 0,02 | 41 | 33 | 74 | 2010 | 21 | 0,003 | 54 | 76 | 130 |  |
| 1972 | 1 | 0,435 | 74 | 2 | 76 | 1982 | 3 | 0,005 | 71 | 62 | 133 |  |
| 1936 | 100 | 0,01 | 27 | 50 | 77 | 1965 | 9 | 0,003 | 64 | 70 | 134 |  |
| 1945 | 119 | 0,008 | 23 | 56 | 79 | 1967 | 4 | 0,004 | 69 | 66 | 135 |  |
| 1948 | 108 | 0,009 | 26 | 53 | 79 | 1980 | 2 | 0,004 | 72 | 67 | 139 |  |
| 1952 | 97 | 0,009 | 29 | 52 | 81 | 1981 | 2 | 0,003 | 73 | 75 | 148 |  |
